# Supplementary figures and images for: teemi: An open-source literate programming approach for iterative design-build-test-learn cycles in bioengineering
Source: PLoS Comput Biol. 2024 Mar 8;20(3):e1011929. doi: 10.1371/journal.pcbi.1011929 (PMC10954146; doi:10.1371/journal.pcbi.1011929)

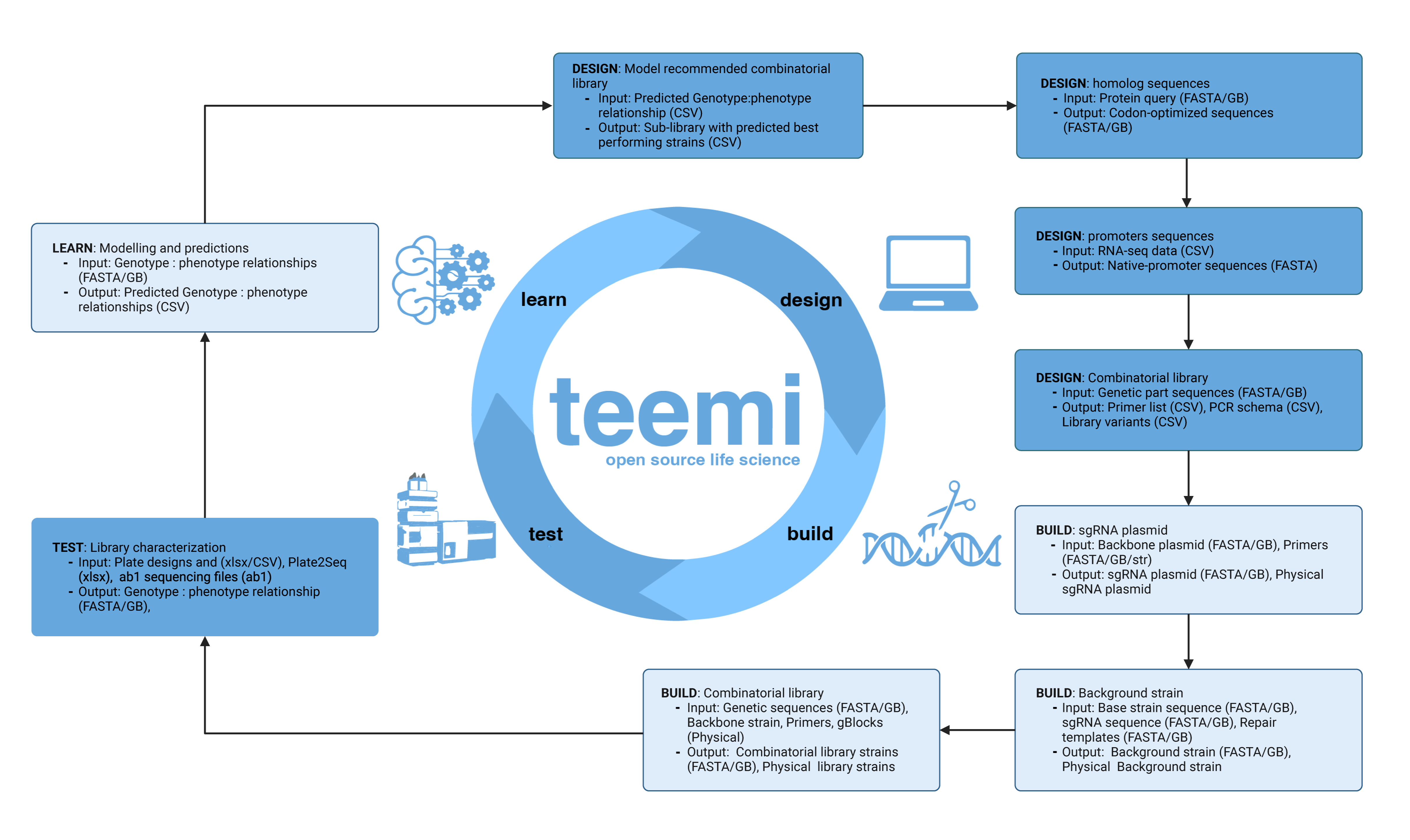

Supplement: S1 Fig — Created with Biorender.com. (TIF) [file pcbi.1011929.s001.tif]

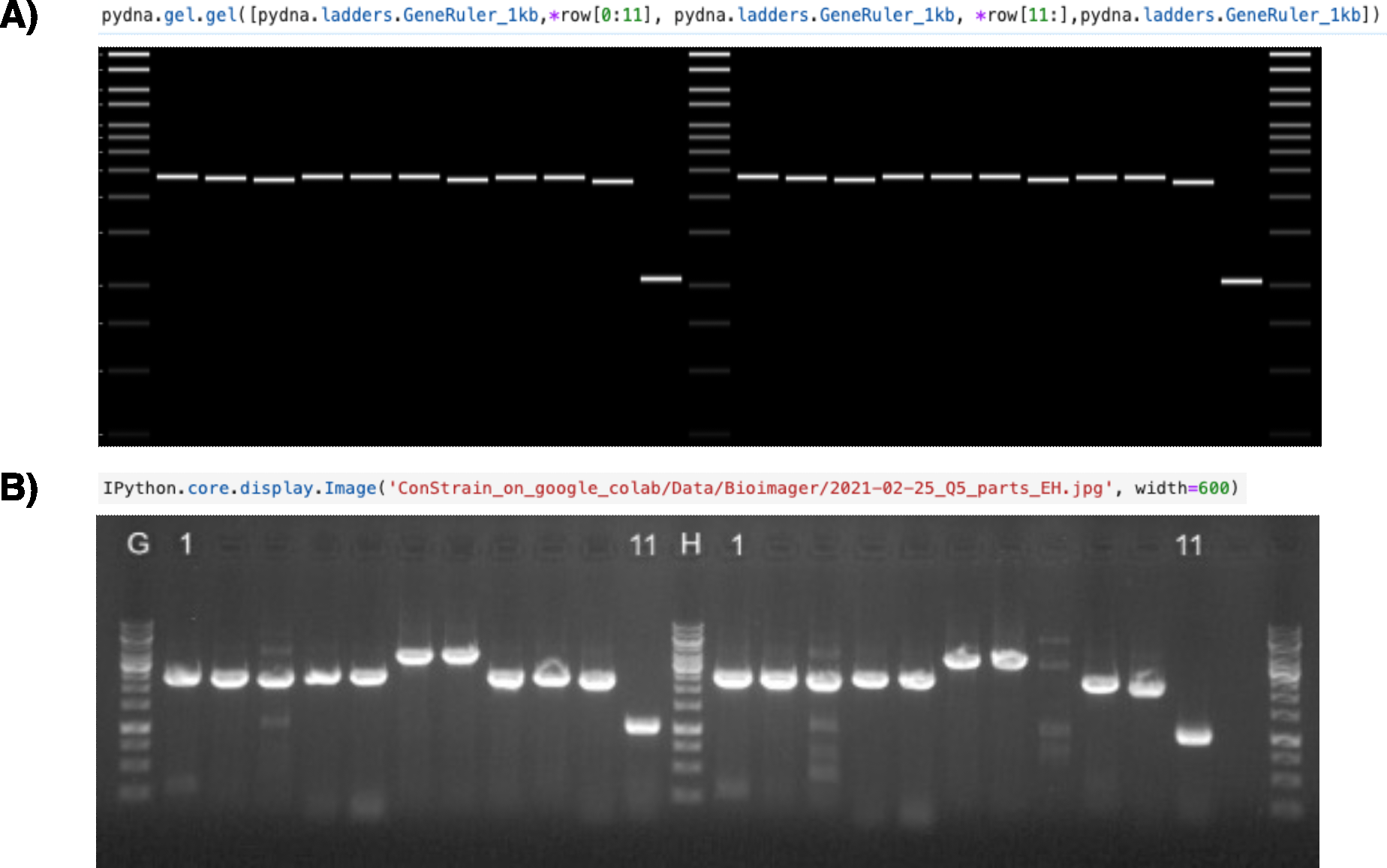

Supplement: S2 Fig — (TIFF) [file pcbi.1011929.s002.tiff]

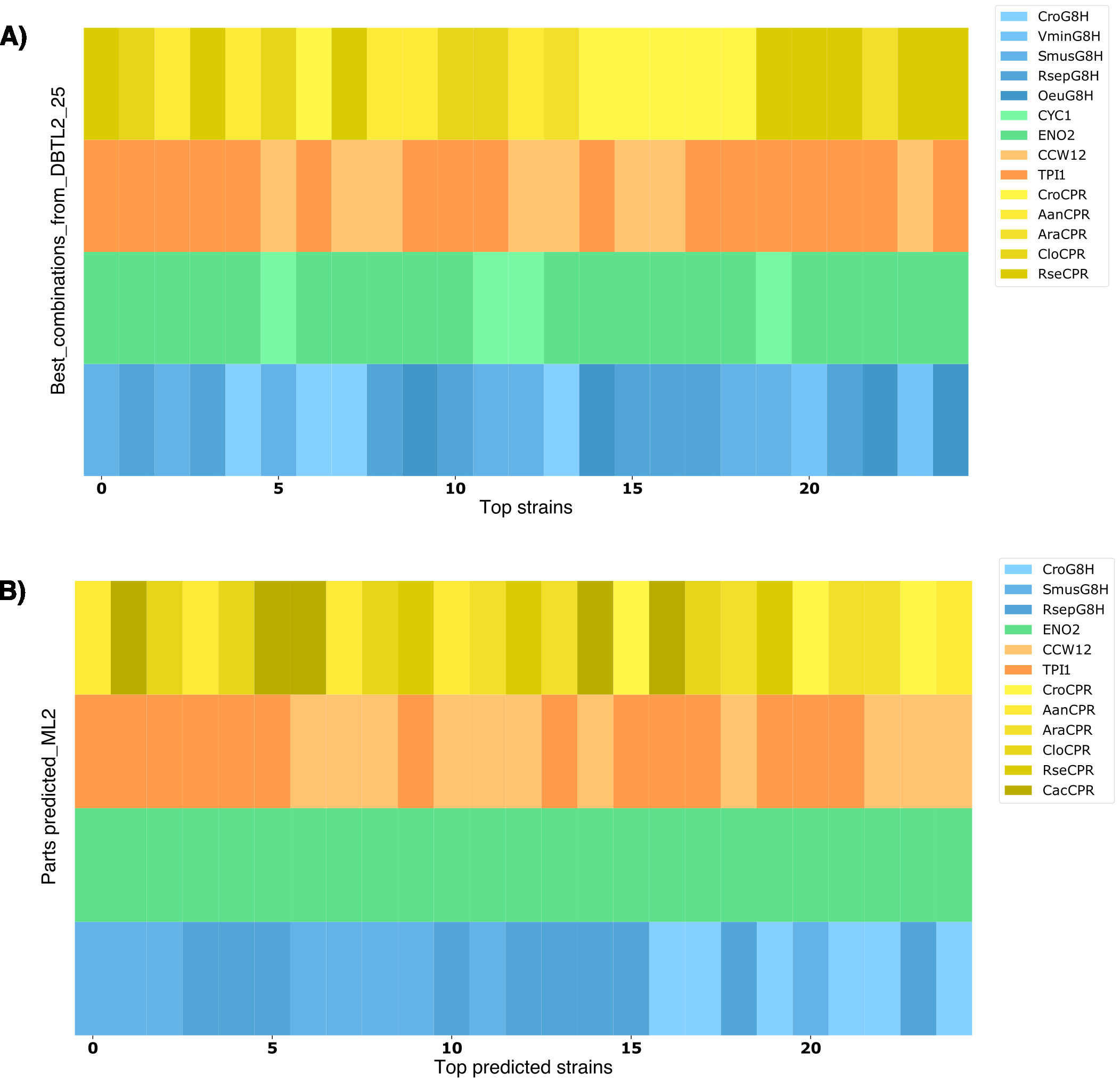

Supplement: S3 Fig — (TIFF) [file pcbi.1011929.s003.tiff]

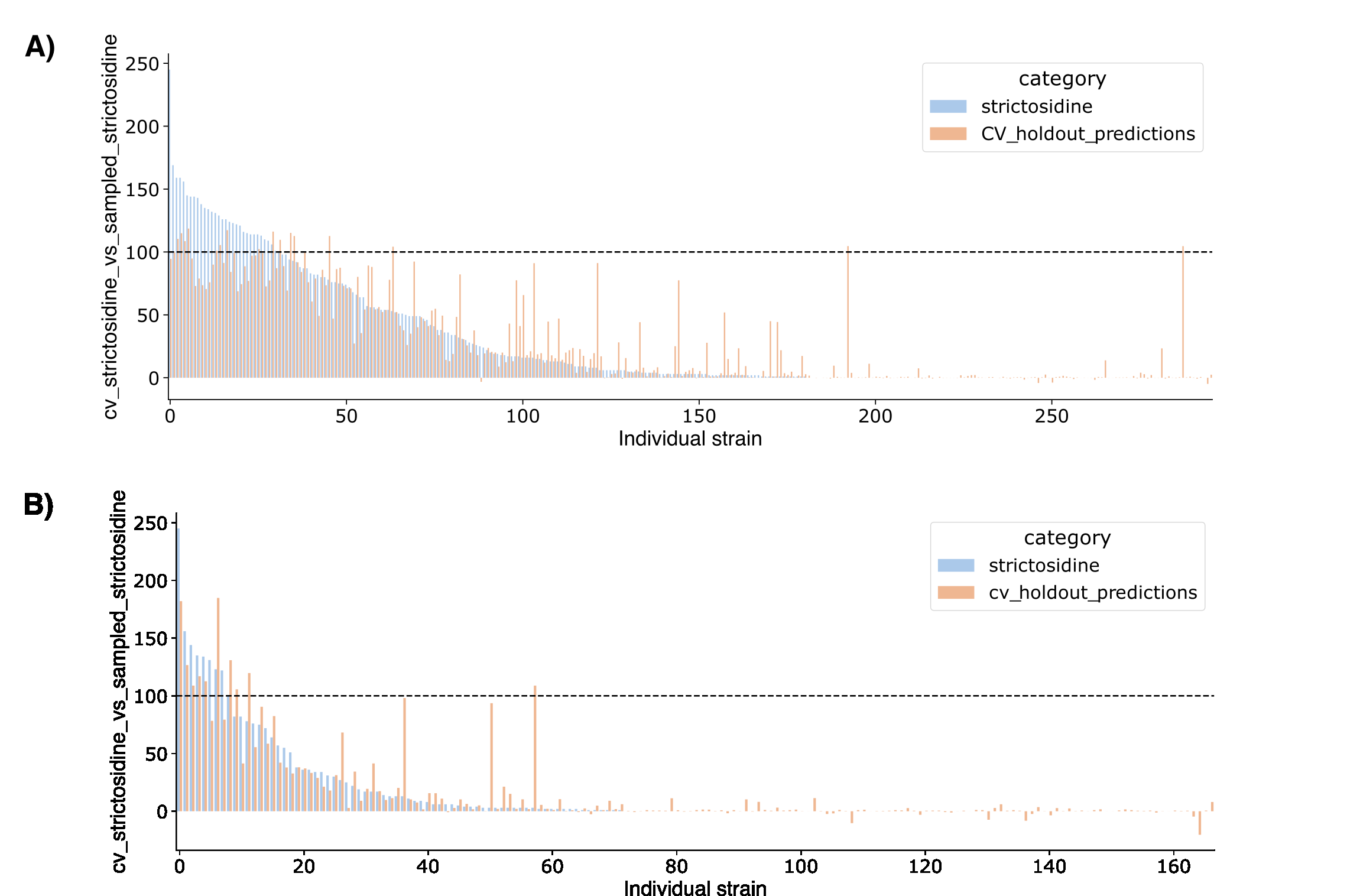

Supplement: S4 Fig — (TIFF) [file pcbi.1011929.s004.tiff]
