# Supplementary material for: teemi: An open-source literate programming approach for iterative design-build-test-learn cycles in bioengineering
Source: PLoS Comput Biol. 2024 Mar 8;20(3):e1011929. doi: 10.1371/journal.pcbi.1011929 (PMC10954146; doi:10.1371/journal.pcbi.1011929)
Supplement: Attachment — Submitted filename: Petersen_Levassor et al_Response to_reviewers.docx [file pcbi.1011929.s008.docx]

**Rebuttal to Reviewers' comments and requests**

We thank the editor and the reviewers for the interest in our manuscript. Based on the comments we have substantially edited the manuscript. We hope the edits, all marked in green, and our detailed point-by-point responses to all comments raised, all marked in red, will be satisfactory to the editor and reviewers, and we look forward to your feedback.

—-------

Reviewer 1: The manuscript describes computational and experimental work which has been executed to a high standard, is novel, interesting and makes a significant contribution to the biotechnology field. As a structural biologist, I am not expert enough in bioinformatics to make an authoritative judgment, but the paper is well-written, technically sound and the standard of presentation is very high. I thought that the introduction was rather brief and would possibly benefit from citing related work of others in this field. Apart from that I am happy to recommend publication of the manuscript in its present form.

>> We thank the reviewer for the positive remarks on our study, and agree with the reviewer’s suggestions for improvements to the introduction. Based on the feedback, we have added the following paragraph to the introduction of the revised manuscript:

“In particular within bioengineering, a literate programming approach has the potential to describe all elements of the DBTL cycle, thus supporting cost-effective laboratory and data analytical workflows. Examples of existing open-source tools that excel in parts of the DBTL cycle include Pydna [21], Aquarium [11], GalaxySynBioCAD [22]. Briefly, Pydna provides descriptions of DNA assembly and cloning strategies in Python with a high degree of flexibility. Complementary to this, Aquarium generates protocols represented as executable code with an integrated LIMS system, while the Galaxy platform [23] offers extensive bioinformatic workflows on genomic analysis through its web-based platform. Extending from this, GalaxySynBioCAD builds on the Galaxy platform with a focus on tools for synthetic biology, such as retrosynthesis and metabolic pathway analyses, through the DBTL cycle. Based on this, there is still a need to provide examples of how tools like these can be assembled into flexible end-to-end DBTL workflows leveraging the best of an ever-increasing palette of tools through literate programming.”

Furthermore, the paragraph includes the following citations: Pereira et al. 2015, Vrana et al. 2021 and Hérisson et al. 2022, Jalili et al. 2020. The citation of Hérisson et al. 2022 is the citation to Galaxy-SynBioCAD that was also sought for by Reviewer #2 in comment #3.

Reviewer 2:

**1.** A workflow of the tool is missing. This omission makes it difficult to gauge the tool's generalizability. To be considered a platform, it should be evident what inputs are required from the user and what outputs the platform provides at each step of the DBTL. Does the user need to modify internal code to use the tool? If so, it resembles a collection of files that could serve as templates or inspiration rather than a platform.

>> We agree that a clearer presentation of the tool's workflow would be an improvement. Therefore we have created a new figure Suppl. Fig. S1 (see below and in revised Suppl. Information) that illustrates the input/output dynamics of each step of the DBTL cycle. This said, teemi is a platform by design. In its first demo presented in this study it includes a minimal collection of templates to execute end-to-end DBTL cycles without the need for further modification. The user is only required to adjust the inputs, which are well-documented in our user guide( <https://teemi.readthedocs.io/en/latest/> ) as acknowledged by Reviewer #2 in comment #6. This said, the ability to fast-track adoption by bioengineers by supporting code modifications pending data type, structure, and learning objectives, is obviously possible in open-source teemi. We hope our response and the updated figure addresses the reviewer’s comment, and that it is clear that teemi stands out as more than a collection of files executed via literate programming.

**2.** The title suggests that the use of literate programming is a significant contribution to DBTL cycles in synthetic biology. However, literate programming is widely used by the synthetic biology community for iterative DBTL, especially through jupyter notebooks. This creates confusion. The title is misleading (the actual name of the package in Github is "a python package designed to make high-throughput strain construction reproducible and FAIR). It is crucial for the authors to clarify the primary contributions of their work.

>> We thank the reviewer for drawing attention to this point of confusion. It is indeed not our intention to claim that we are the only ones using jupyter notebooks and literate programming. To clarify this we have now:

1. changed the title from "Literate programming for iterative design-build-test-learn cycles in bioengineering" to "teemi: An open-source literate programming approach for iterative design-build-test-learn cycles in bioengineering"
2. added a paragraph in the Introduction further specifying the primary contribution of this work - please see our response to Reviewer #1, Comment #1)
3. changed to name of the Github package to "teemi: An open-source literate programming approach for iterative design-build-test-learn cycles in bioengineering"

**3.** Table 1 is notably incomplete. Please update it with platforms that have already been released and published, addressing the DBTL cycle. For example, the Galaxy project is cited, but what about GalaxySynbioCad, which is a Galaxy-based platform specifically tailored to the DBTL pipeline? (<https://www.nature.com/articles/s41467-022-32661-x>)

>> This is a good point. We overlooked this reference, and it of course needs to be cited in this table summarizing prior art. Indeed, referencing the GalaxySynBioCad fits well with the motivation and the conclusions of our study, and we have now included it in our Table 1. Furthermore, and as responded to the Reviewer’s comment #2, we have also added GalaxySynBioCAD and other relevant tools to the revised Introduction, to put emphasis on the motivation for the development of teemi.

Lastly, we would like to mention that we have refined the scope of Table 1 to focus exclusively on maintained open-source tools that are directly applicable to the DBTL framework to be more relevant for the scope of teemi.

**4.** The main assumptions, decisions, and methods adopted at each stage of the DBTL should be clearly explained (in connection with comment 1). For instance, in the BUILD module, the authors adopted CasEMBLR for CRISPR/Cas9-mediated assembly, and in the TEST stage, they used DMA Sequence and LC-MS...

>> This is a valid point, especially considering the scope of the journal and the diverse readership. Decisions for choice of methods have now been updated for each of the sections describing the DBTL cycle. The list of these changes made to the revised manuscript is presented below:

For the Design step we updated the revised manuscript with this paragraph:

“The first iteration of the teemi-based DESIGN module, under the DBTL framework, focused on enzyme homology searches, promoter choices, and primer designs. This approach aimed to leverage the extensive and increasing genomic resources available in databases like NCBI (Ref). Using a top-down approach is particularly useful when the enzymatic pathway is known. Building on this, we developed an algorithm to standardize screening and selection of homologs (00_1_DESIGN_Homologs, Paragraph: 1) using Catharanthus roseus G8H and CPR sequences as queries [23,25].”

For the BUILD step we updated the revised manuscript with this paragraph:

“The choice of method used for the BUILD step, focused on reducing potential heterogeneity of the designed expression units and the number of wet lab steps. With CasEMBLR this would be enabled by stable genomic integration of assembled expression clusters as defined in the DESIGN step and direct transformation of parts rather than the adoption of shuttle vectors propagated via a bacterial host, respectively. For this purpose, CasEMBLR is a well-suited tool as it leverages high-fidelity multi-part assembly based on homologous recombination and limits expression stochasticity by genomic integration of the assembled expression units.”

For the TEST step we updated the revised manuscript with this paragraph:

“The choice on which data to capture in the TEST module was guided by simple principles. First, working on engineering biology, bioengineers are often aiming to correlate, and even predict, a sought-for phenotype of a living system based on a given genotype. While genotype is largely described by DNA sequencing, the phenotype of interest can be assessed by a multitude of parameters and proxies, such as fitness, shape, taste, and function. In this study, the objective function was to adopt teemi for the cost-effective engineering of cell factories and the prediction of production titers in engineered cell factories based on their respective genotypes. As such, the minimal set of input data to be used for model-guided optimization of cell factories would be genotype and titers based on Sanger-based DNA sequencing and quantitative liquid chromatography-mass spectrometry data, respectively. ”

For the LEARN step we updated the revised manuscript with this paragraph:

“Commonly used machine learning models include XGBoost models which have previously been shown to have high predictive capabilities in modeling gene expression values [38], as well as genetic and metabolic networks [39]. Complementary to this, DeepLearning algorithms have been used in a plethora of bioengineering applications notably to predict guide-RNA activity for CRISPR/Cas-based genome engineering [40] and gene regulation [41]. Additionally, stacked ensembles, distributed random forest (DRF), general linear models (GLMs), and gradient booster models (GBM) models have also found applications in various biological areas [42][43]. Taken together, the adoption of such a vast repertoire of computational methodologies emphasize the diverse degree of complexity found in nature and biological research. However, as no single machine learning algorithm is optimal for all learning tasks [44,45], 1,895 different models, including DRF, GLM, XGBoost, GBM, DeepLearning, and StackedEnsemble sourced from H2O AutoML [46],, were made as a function of gene and promoter combinations combined with normalized strictosidine measurements (input_for_ml.csv).”

We hope these additional paragraphs clarifies our rationale for the methods and approaches adopted in this study, and that they as such respond satisfactorily to the reviewers point.

**5.**  In the LEARN stage, the authors claim that the library for the next iteration is based on the top-performing predicted models. However, it seems that no combinatorial experimental design is applied to enhance the exploration of solutions. This could introduce bias into the results, and this aspect is not adequately clarified in the text.

>> OK. When we received the predicted designs from the top prediction models following the first round of DBTL cycle, we created these designs in a pooled library construction instead of creating each strain individually. We did this as this would enable us to build more strains per time, which together with stochastic sampling would expedite the TEST step while at the same time maximize exploitation of the given design space. We designed the library by taking the best genetic parts predicted from the models (cf. [08_2_DESIGN_Model_recommended_combinatiorial_library.ipynb](https://colab.research.google.com/github/hiyama341/teemi/blob/main/colab_notebooks/08_2_DESIGN_Model_recommended_combinatiorial_library.ipynb)). This does introduce a bias as the new model doesn't get data from the exact top predicted strains but from more strains among the top predicted strains. We have elaborated on this decision in the section “*teemi for design-build-test-learn cycle II*”:

“Balancing the maximum remaining search space (1,121 designs) and the predictive power of the deep learning model trained on data from the first DBTL cycle (159 designs), we decided for a maximum build capacity of 180 strain designs based on the parts found among the 20 predicted top-performers. This resulted in a distribution of 5 G8Hs, 2 promoters for controlling expression of genes encoding G8Hs, 2 promoters for controlling expression of genes encoding CPRs, and 7 CPRs (16 parts in total, creating a theoretical combinatorial space of 140 strains)(08_2_DESIGN_Model_recommended_combinatiorial_library, Paragraph: 1)(Fig. 4A). Based on these parts, the combinatorial optimization approach was conducted as in the first DBTL cycle (Fig. 3A). Of note, by choosing this combinatorial optimization approach we were biased towards “generating more hits-on-target” rather than the exactness of the constructed genotypes. This said, among the top-20 designs investigated, that form the basis of our sublibrary in the second DBL cycle, 16 of the highest predicted designs were made (16/20= 80%), indicating only a modest trade-off between exploration and exploitation within the constrained design space of this study.

In addition to this we added a perspective in the Discussion, to highlight the fact that extending the search space, incl. various complementary tools to support diversity generation, would be a next logical step to further increase strictosidine titers. Importantly, this extension of the design space would be perfectly compatible with the existing features and functionalities of teemi. The paragraph inserted in the Discussion is:

“To generate top-performing strains requires high-performing genetic parts and finding top-performing genetic parts is a hurdle when constructing biosynthetic pathways. Here we used BLAST to find homologs to catalyze our challenging hydroxylation step, but an alternative approach would be to BLAST and cluster sequences with, for example, the open-source tool MMseqs2 [48] and introduce variants from each cluster to increase library diversity. Another way to find alternative genetic parts, circumvent bottlenecks in the pathway, or find novel biosynthesis routes would be to use retrobiosynthesis tools like Retropath 2.0 [49] or the BNICE framework [50]. These strategies align with the iterative nature of the DBTL cycle, where each step is crucial for efficient pathway engineering.”

**6.** The tool's features and functionalities need to be specified. The term "engineering of complex biosystems" is too abstract. The features supported are clearly specified in the documentation of the Python package, but not in the paper.

>> We thank the reviewer for complimenting the clarity of the feature specification in our python package and for pointing out that the tools could be better specified in the paper. As mentioned in response to reviewer #2's comment #1, we have now created a new figure Suppl. Fig. S1 that more clearly illustrates the specific features and functionalities of the tool in the paper. We believe that this figure nicely complements the detailed explanations available in the Python package documentation.

Furthermore, we have removed the word “complex” in relation to biosystems in the Abstract as well as in the main text.
